# Advantages of the Application of the Temper Bead Welding Technique During Wet Welding

**DOI:** 10.3390/ma12060915

**Published:** 2019-03-19

**Authors:** Jacek Tomków, Grzegorz Rogalski, Dariusz Fydrych, Jerzy Łabanowski

**Affiliations:** Faculty of Mechanical Engineering, Gdańsk University of Technology, G. Narutowicza street 11/12, 80-233 Gdańsk, Poland; grzegorz.rogalski@pg.edu.pl (G.R.); darfydry@pg.edu.pl (D.F.); jerzy.labanowski@pg.edu.pl (J.Ł.)

**Keywords:** underwater welding, weldability, temper bead welding, cold cracking, wet welding, CTS test, covered electrodes, hardness measurements, steel

## Abstract

Thermo-mechanically rolled S460ML steel was chosen for welding in underwater wet welding conditions by covered electrodes. The main aim of this study was to check the weldability for fillet welds in a water environment by controlled thermal severity (CTS) tests and to check the influence of temper bead welding (TBW) on the weldability of the investigated steel. Non-destructive and destructive tests showed that S460ML steel has a high susceptibility to cold cracking. In all joints, hardness in the heat-affected zone (HAZ) was extended to the 400 HV10 values. Microscopic testing showed the presence of microcracks in the HAZ of all welded joints. TBW was chosen as the method to improve the weldability of the investigated steel. This technique allows for the reduction of the maximum hardness in the HAZ below the critical value of 380 HV10, as stated by the EN-ISO 15614-1:2017. It was determined that for S460ML steel, from the point of view of weldability, the pitch between two beads should be in the range 75%–100%. Also, if the pitch between two beads increases, the hardness, grain size, and number of cracks decreases. In all specimens where the hardness of the HAZ was below 380 HV10, there were no microcracks.

## 1. Introduction

The research into the processes of joining in underwater conditions is developing annually. There are many studies on the use of friction stir welding (FSW) in this environment [1,2]. FSW produces a small heat-affected zone (HAZ), which creates better mechanical properties in joints [3]. However, the most popular method of joining is still arc welding. There are three methods of welding in underwater conditions. The first is dry welding, in which the welder and welding area are in a specialized chamber without contact with the aqua environment [4,5]. The second method is welding with the use of a local dry chamber. In this method, the diver/welder is in direct contact with the water, but the welding area is located in a special chamber that isolates this area from the surrounding water environment [6,7]. The final and most common method of underwater welding is wet welding, where the diver and welding area are in contact with the water environment. This method is the cheapest and can be used for many applications, including offshore and marine structures, which may require repairs underwater [8].

Repair welding in the open air is a well-known process and often results in joints with good mechanical properties. Welding in an underwater environment, on the other hand, generates some significant problems due to imperfections and the lower mechanical properties of underwater joints in comparison to joints made in the open air. The weldability of steel joints made in open air conditions is often better than those made in the water [9,10].

Water as a welding environment causes instability of the electric arc. However, there are some studies in this field that have showed that stability could be improved by using a static mechanical constraint support [11]. The other method that could improve the stability of the welding arc is welding with filler materials with a calcium fluoride content [12].

There are three big problems generated by the underwater conditions. Firstly, the high cooling rate, which makes the t_8/5_ (cooling time in the temperature range 800–500 °C) much shorter than in air welding [13]. This leads to the formation hardened structures in the HAZ. The second problem is the presence of residual stresses after welding. The third factor that generates the lower quality of underwater joint is the diffusible hydrogen content in deposited metal [14]. These three factors are responsible for the susceptibility to cold cracking, which is the biggest problem in underwater wet-welding processes [15,16]. Cold cracks can be located in the weld and in the HAZ. Cold cracking is the main problem in the welding of high-strength low allow (HSLA) steels [17], which are commonly used as a part of offshore constructions [18]. There are a few methods for improving the weldability of steels that are commonly used for joints made in the open air. The water environment precludes the use of most of them. For air welding, one of the typical methods for improving the weldability of steel is the pre-heating of the base material during welding. The latest laboratory experiments show that induction heating in the water is possible [19], however, this technique is still not well-known and is not used in real structures.

An alternative solution that offers the possibility of controlling the thermal welding cycle is temper bead welding (TBW) [20,21]. It consists of the application of additional beads inside an existing weld or on its face layer. The area of application of this technique mainly involves air repair welding using the MMA, TIG and FCAW processes in the power [22] and pressure equipment industry [23]. TBW provides local heat treatment to prior weld layers and their HAZ. The heat of the tempering beads can refine and temper the coarse-grained HAZ of the existing welds. It can provide a reduction in hardness in the HAZ [24]. At the Gdańsk University of Technology, research was carried out to show the possible effectiveness of TBW for steels from other groups than those investigated in this article [25,26]. The studies of the TBW technique show that for S355G10 + N and S460N steel, TBW can be an effective method to limit the susceptibility to cold cracking.

The thermo-mechanically rolled steels have better weldability than normalized, quenched and tempered steels [27]. However, the behavior of the thermo-mechanically rolled steels during wet welding conditions requires testing.

The first aim of this research was to check the weldability of thermo-mechanically rolled S460ML steel (Ce_IIW_ = 0.365%) in underwater conditions using wet welding method for joints welded with covered electrodes. The weldability of this steel is well known, and joints made in open air using this steel are of good quality [28]. The second aim of this study was to investigate the relationship between the structure and the hardness of the HAZ for the different values of the pitch during wet TBW. This has not been reported in the literature to date.

## 2. Materials and Methods

### 2.1. Materials 

For the welding, S460ML 14 mm-thick plates were chosen. As a filler material, ISO 2560-A: E 38 0 R 11 [29] rutile electrodes with a diameter of 4.0 mm were used. These produce good plasticity of the welds, which can reduce the susceptibility to cold cracking. The chemical composition of the S460ML steel and electrodes is listed in Table 1. Table 2 presents the mechanical properties of the used materials.

### 2.2. Welding Process

**Figure 1:**
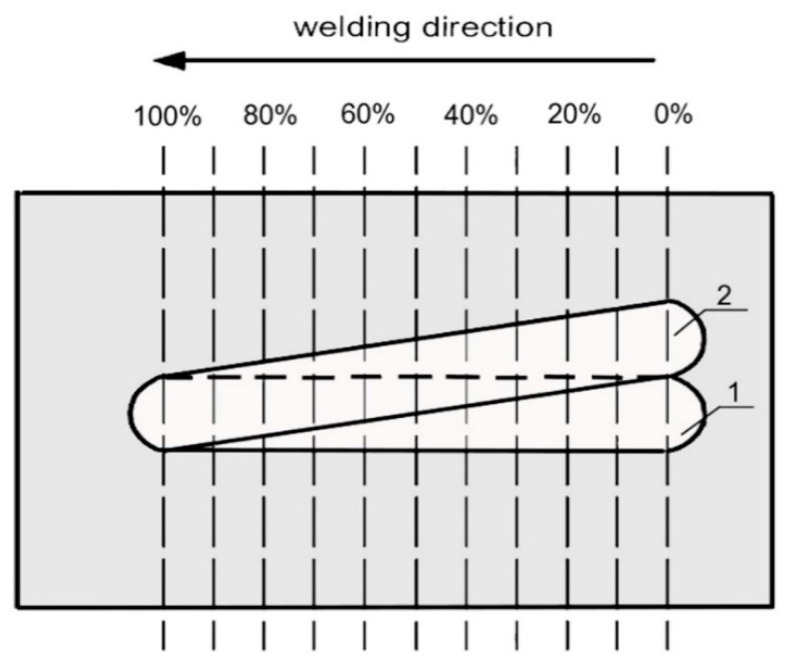
Scheme of the temper bead welding (TBW) technique specimen, 1—first (tempered) padding weld, 2—second (tempering) padding weld.

There are many weldability tests, for example the controlled thermal severity (CTS), Tekken, and implant tests [30,31,32]. For this study, the CTS test was chosen, because in a water environment the most popular welding method uses fillet welds. In addition, this test enables assessment of the susceptibility to cold cracking in all joint areas. In the next step, the TBW technique was used for pad welding to determine the influence of the pitch on the microstructure of the HAZ and the hardness of the joints. TBW specimens were made by varying the relative deposition of the beads according to methodology presented in the literature [25]. Weld beads for the TBW samples were made on 200 mm × 100 mm sections in a flat welding position. The welds were laid in order to obtain different distances between the axes of the beads (in a non-parallel manner). The welding schema with a temper bead is presented in Figure 1. This method allowed us to obtain specimens with different percentages of overlap of the second weld bead onto the first weld (pitch). Between two welds, 120 s time passed. All samples were made at a depth of 0.5 m. The diffusible hydrogen content in the deposited metal obtained by the glycerin method for wet welding with E 38 9 R 11 electrodes were in the range of 51.5 to 61.4 mL/100 g [33]. 

The CTS specimens were made in accordance with the EN ISO 17642:2005 [34] standard with the parameters given in Table 3. The TBW specimen parameters are shown in Table 4.

### 2.3. Examination Procedure

After the welding process, all specimens were tested using visual testing (VT) and penetrant testing (PT), as well as metallographic macroscopic and microscopic testing. Then, the Vickers HV10 hardness measurements were taken in accordance with EN ISO 9015-1:2011 [35]. The investigated S460ML steel was classified as a material from the material group 2.2 in accordance with the EN-ISO 15614-1:2017 [36]. The maximum hardness values of the HAZ recommended by this standard cannot exceed the level of 380 HV10.

**Figure 2:**
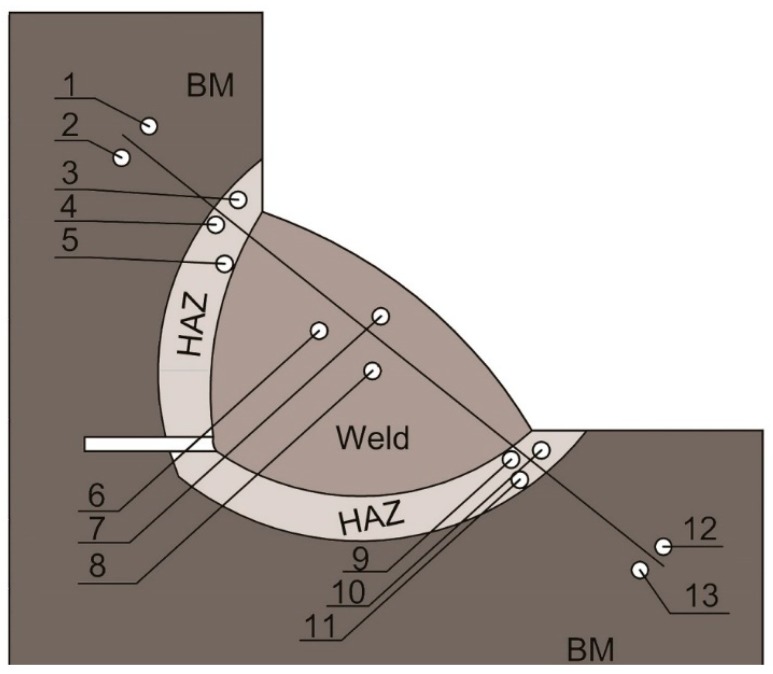
Hardness point distribution in the CTS specimens.

**Figure 3:**
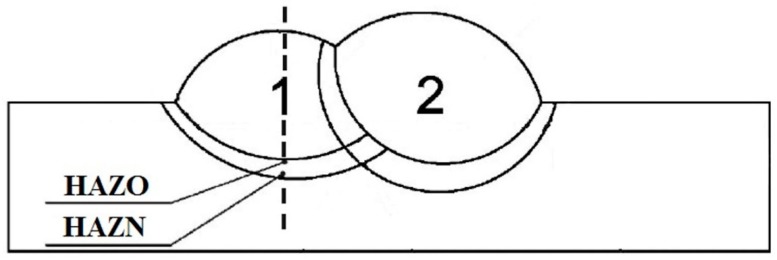
Hardness measurement areas of the heat affected zone (HAZ)—TBW specimens, overheated area of HAZ (HAZO) of the 1st padding weld in the weld axis, normalization area of HAZ (HAZN) of the 1st padding weld in the weld axis.

The hardness measurement scheme for CTS testing is presented in Figure 2. Figure 3 shows the regions of the TBW specimens in which the hardness was measured. The hardness measurements in the first padding weld of the normalization area of HAZ (HAZN) and the overheated area of the HAZ (HAZO) were located in the HAZ at the axis of the first bead. 

## 3. Results and Discussion

### 3.1. CTS Test

#### 3.1.1. Microscopic Testing

**Figure 4:**
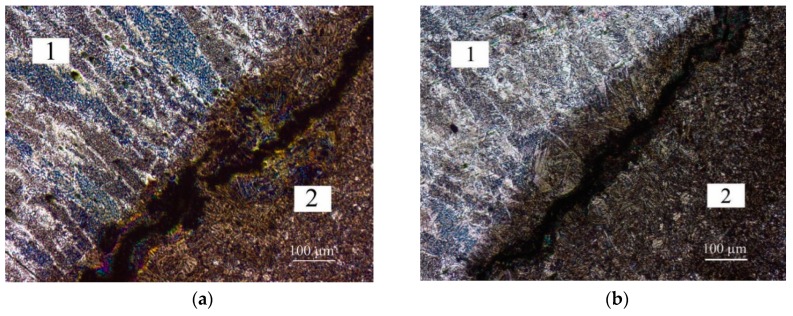

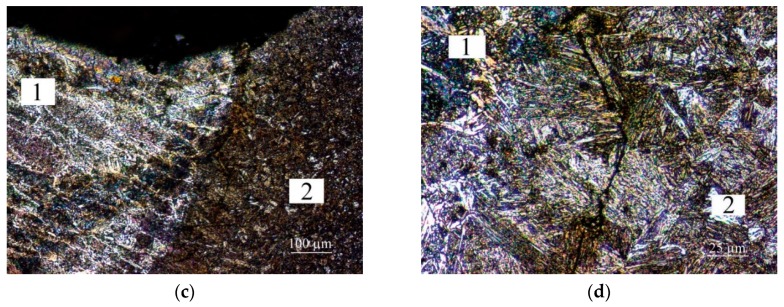
Results of CTS microscopic testing shows cracks in HAZ, 1—weld metal, 2—HAZ, (**a**) Specimen 1; (**b**) Specimen 2; (**c**) Specimen 3; (**d**) Specimen 6. Etch. Nital 4%.

After non-destructive testing, second welds from specimens 1, 2, 5 and 6 were rejected from further examinations due to significant imperfections. The remaining welds were capable of undergoing further examinations. In the HAZ of each specimen, acicular and martensitic structures were found, which are a result of rapid cooling during welding in the water. The short cooling time also caused high residual stresses and limited the extraction of diffusible hydrogen in the welding environment. The weld metal structure was built of dendrites. The results of the microscopic examination of CTS specimens are presented in Figure 4. During macroscopic testing, cracks in the HAZ of the two specimens (the first weld in specimen 4 and specimen 6) and in weld of one specimen (first weld in specimen 4) were observed. The cracks in the HAZ were initiated in the weld root and propagated along approximately 50% of the length of the fusion line. This area was characterized by an overheated, coarse grain, martensitic structure as is shown in Figure 4a,b. The cracks were observed in the welded joints that were made with the lowest value of heat input (*ql*). Microscopic examination of S460ML steel showed that cracks occurred in all joints welded in the water environment. These cracks were located in the HAZ, along the fusion line. It was estimated that the total length of these cracks were 30%–40% of the total length of the fusion line. Cracks in the weld also were revealed.

#### 3.1.2. Hardness Measurements

The hardness measurements were performed on cross-sections of at least one weld from each CTS specimen. The results of the hardness measurements are presented in Table 5.

The CTS tests showed that the weldability of the S460ML steel in underwater conditions can be described as poor. For all specimens, the maximum HV10 hardness in the HAZ exceeded the critical value of 380 HV10. This proved that in this area of each specimen, brittle martensitic structures formed. There were also many branched microcracks in the welded joints.

### 3.2. Temper Bead Welding Test

#### 3.2.1. Macroscopic Testing

**Figure 5:**
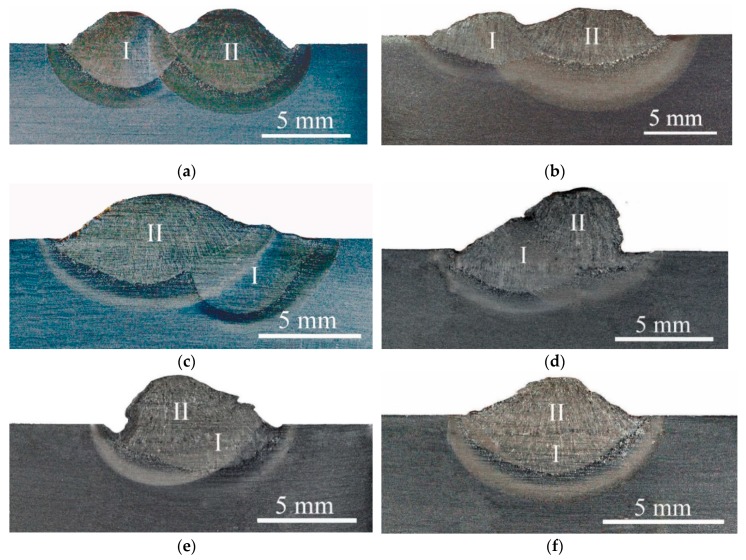
Results of the TBW macroscopic testing, I—first (tempered) bead, II—second (tempering) bead, (**a**) pitch 11%; (**b**) pitch 19%; (**c**) pitch 43%; (**d**) pitch 55%; (**e**) pitch 73%; (**f**) pitch 100%. Etch. Nital 4%.

In the first step of the examination, macroscopic tests were performed. The main aim of these examinations was to calculate the pitch between the two beads. After the macroscopic testing of specimens with a pitch of; 11%, 19%, 43%, 55%, 73%, and 100%, were selected for the next examinations. There were no cracks in these specimens. The results of the macroscopic testing are presented in Figure 5.

#### 3.2.2. Microscopic Testing

**Figure 6:**
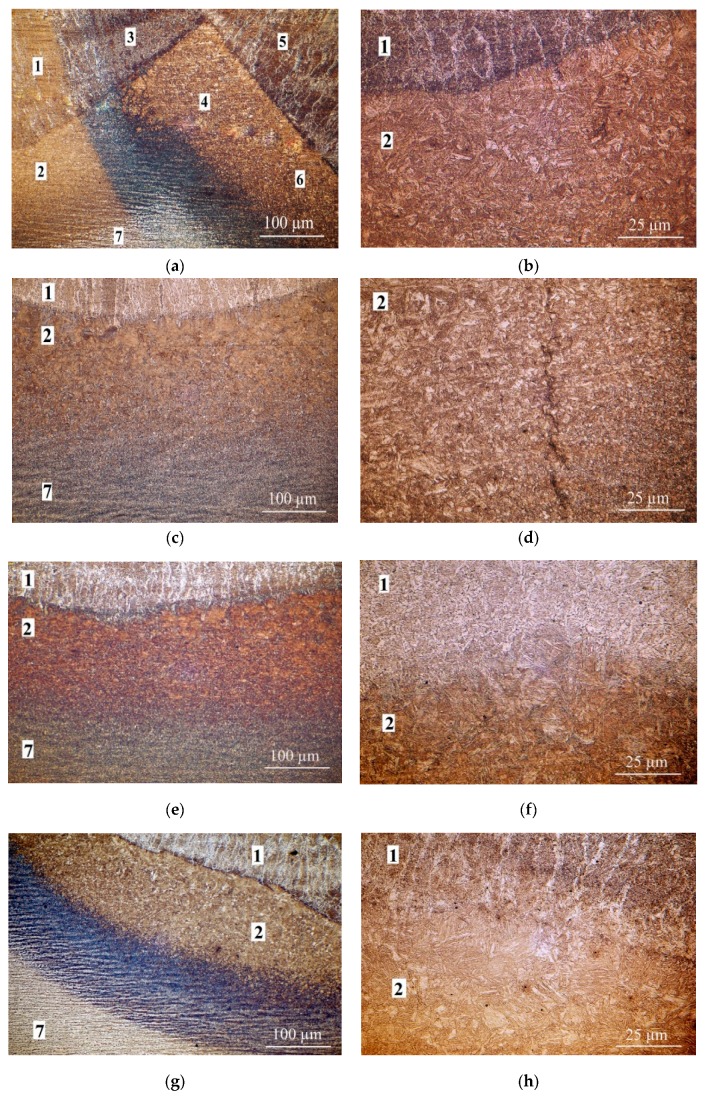

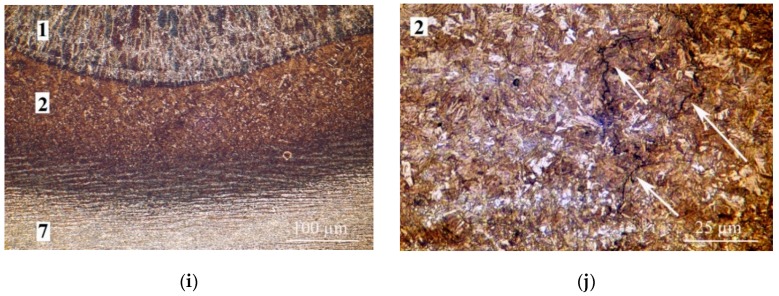
Results of TBW microscopic testing; 1—first bead, 2—HAZ under first bead, 3—area of the HAZ of the second bead overlapping the first bead, 4—HAZ of the second bead overlapping the HAZ of the first bead, 5—second bead, 6—HAZ under the second bead, 7—base metal. (**a**,**b**) pitch 11%; (**c**,**d**) pitch 43%; (**e**,**f**) pitch 55%; (**g**,**h**) pitch 73%; (**i**,**j**) pitch 100%. Etch. Nital 4%.

The results of the microscopic testing are presented in Figure 6. The welds in each of these specimens showed dendritic structures (Figure 6a, area 1). There was bright, fine-grained ferrite arranged in columns, from which grew acicular ferrite at the boundaries of the dendrites. Inside the dendrites there were fine ferrite grains. The structure of the HAZ was different. Near the fusion line, there were expanded quasiperlite grains with a structure following the Widmannstätten system (e.g., Figure 6b,c, area 2). The structure of this zone also indicated the presence of bainitic hardening structures and low-carbon martensite (Figure 6d). In the areas located the furthest distance from the fusion line, there were normalization structures with fine ferrite and pearlite. In all specimens, changes due to the tempering bead were observed. With the highest value of the pitch between two beads, there was a partial disappearance of the structure of the dendritic base bead and the formation of a ferritic fine-grained structure (e.g., Figure 6e,f, area 1). In the HAZ, the heat of the tempering beads refined and tempered the coarse-grained zone of the first bead (Figure 6g). When the pitch increased, the grain size decreased. TBW provided local heat treatment of the joints. The maximum hardness values for wet welding appeared in the coarse grained HAZ [37]. The grain size decreased during TBW (Figure 6h,i, compare with Figure 6a–d), which led to a reduction of hardness in the HAZ. The pitch increased and the number of cracks decreased. However, TBW could not repair the microcracks that occurred during the welding of the first bead and could even propagate these cracks (Figure 6j). Cold cracks can occur up to 48 h after welding is completed. TBW was used in the 120 s after first bead was laid, thus this technique reduced the probability of the occurrence of these cracks.

#### 3.2.3. Hardness Measurements

**Figure 7:**
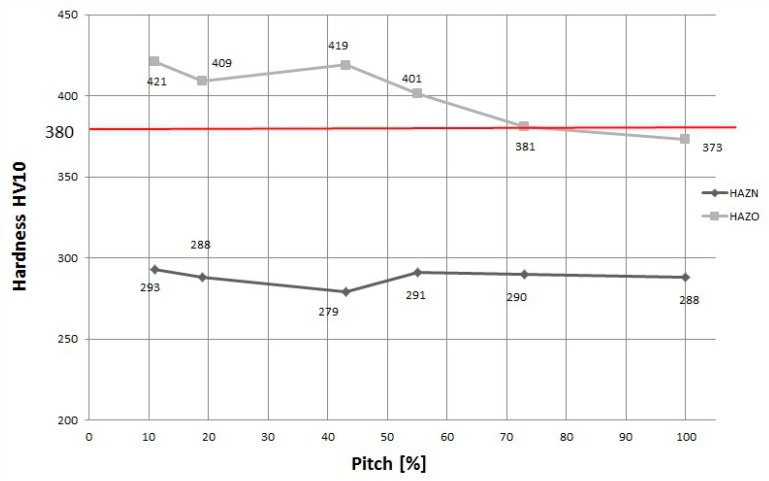
Hardness distribution for specimens with different overlap values in HAZO and HAZN areas of the first (tempered) weld.

The hardness measurements were performed on cross-sections of each specimen with all ranges of pitch. The hardness was measured at points in two specific areas of the HAZ (overheated and normalized) of the first tempered weld (Figure 3). The results of the hardness measurements are presented in Figure 7. When the pitch between two beads increased, the hardness in the HAZO of the first padding weld decreased. The HAZO area in the specimens with low pitch values was characterized by the presence of the coarse-grained brittle structures created by the highest cooling rate in the welded joint (Figure 6b). These types of structures are one of the factors responsible for cold cracking. Thus, the HAZO area has a crucial influence on the formation of cracks.

In all CTS specimens the hardness was greater than 405 HV10. The HAZ of the tempered beads showed a significant reduction in hardness after using the TBW technique. For specimens with a pitch in the range of 75%–100% the hardness values were below 380 HV10 in the overheated area. In the HAZN, no significant changes in hardness were observed. The comparison in hardness decreased in the HAZ for different steels, as presented in Table 6. The results showed that the TBW technique was more effective for steel with a Ce_IIW_ lower than 0.4%.

## 4. Conclusions

The results of this experiment showed that the cracking of S460ML joints made in underwater conditions occurs due to a typical mechanism used when welding steel with a higher carbon content than the filler material. This phenomenon causes hydrogen diffusion from the weld to the HAZ. The influence of residual stress on brittle structures saturated with hydrogen results in cold cracking.

The conclusions drawn based on the experiment results and discussion are:

- S460ML thermo-mechanically rolled steel has high susceptibility to cold cracking in wet welding conditions using covered electrodes. The joints are characterized by high hardness (above the critical value of 380 HV10). There is also a lot of cracking in the HAZ.
- The TBW technique allows for reducing hardness in the HAZ of specimens made in the water environment. In comparison to the CTS test specimens, the hardness after TBW decreased by 63 HV10. The TBW technique could also reduce number of microcracks in comparison with the CTS test specimens.
- When the pitch between two beads increases, the hardness, grain size and the number of cracks decreases. However, TBW is ineffective for repairing cracks that occurred while the first bead was welded.
- From a weldability point of view, the best pitch for S460ML thermo-mechanically rolled steel welded underwater is above 75%. Above this pitch the hardness is lower than the critical value 380 HV10.

## Figures and Tables

**Table 1:** Chemical composition of used materials wt.%.

Material	C	Si	Mn	P	S	Cr	Mo	Ni	Cu	V	Ce_IIW_ *
S460ML in accordance with control analysis	0.12	0.50	1.40	0.015	0.004	0.04	0.01	0.02	0.01	0.01	0.365
E 38 9 R 11 electrodes deposit in accordance with manufacturer data	0.07	0.44	0.55	0.01	0.02	0.04	–	–	0.05	–	–

* Ce_IIW_—carbon equivalent by the International Institute of Welding.

**Table 2:** Mechanical properties of used materials in accordance with manufacturer data.

Material	*R_e_* (MPa)	*R_m_* (MPa)	*A*_5_ (%)
S460ML	515	598	25
E 38 0 R 11 electrodes deposit	503	538	26

**Table 3:** Welding parameters for the controlled thermal severity (CTS) tests.

Specimen No.	*I* (A)	*U* (V)	*t* (s)	*V*sp (mm/s)	*ql* (kJ/mm)	*I* (A)	*U* (V)	*t* (s)	*V*sp (mm/s)	*ql* (kJ/mm)
	First Weld					Second Weld				
1	188	23.0	18.8	3.99	1.08	188	23.8	14.9	5.03	0.89
2	188	23.8	18.5	4.06	1.10	180	26.0	14.7	5.14	0.91
3	188	22.5	19.5	3.85	1.10	184	24.3	18.9	3.95	1.12
4	188	22.8	12.8	5.86	0.73	184	25.0	18.0	4.16	1.11
5	180	25.2	17.5	4.28	1.07	192	21.8	15.4	4.87	0.86
6	184	24.3	16.5	4.55	0.98	188	23.5	19.3	3.88	1.14

**Table 4:** Welding parameters for the TBW technique.

Specimen No.	Pad Weld	*I* (A)	*U* (V)	*T* (s)	*L* (mm)	*V*sp (mm/s)	*ql* (kJ/mm)
TBW1	1	192	25.5	17.6	121	6.88	0.71
	2	204	29.0	17.0	125	7.35	0.80
TBW2	1	188	27.5	16.1	130	8.07	0.64
	2	204	30.5	14.9	125	8.39	0.74
TBW3	1	188	27.3	18.9	136	7.20	0.71
	2	208	27.5	16.8	130	7.74	0.74
TBW4	1	188	26.3	17.0	115	6.76	0.73
	2	205	27.3	14.7	120	8.13	0.70
TBW5	1	188	27.3	17.9	147	8.21	0.62
	2	205	28.0	13.3	92	6.92	0.84

**Table 5:** Results of hardness measurements—CTS specimens.

Specimen No.	Hardness HV10													*ql* (kJ/mm)
	BM		HAZ			Weld			HAZ			BM		
Measurement point	1	2	3	4	5	6	7	8	9	10	11	12	13	
1														
First weld	230	221	390	376	380	276	299	258	405	405	380	221	224	1.08
Second weld	–	–	–	–	–	–	–	–	–	–	–	–	–	0.89
2														
First weld	224	228	425	432	402	272	254	278	423	400	418	224	221	1.10
Second weld	–	–	–	–	–	–	–	–	–	–	–	–	–	0.91
3														
First weld	219	218	397	422	412	272	264	253	403	380	397	208	209	1.10
Second weld	223	218	391	433	421	233	254	260	406	380	425	211	221	1.12
4														
First weld	204	222	377	397	380	292	281	285	400	400	421	208	232	0.73
Second weld	230	217	411	417	427	254	240	239	392	389	405	218	209	1.11
5														
First weld	215	215	419	439	424	278	261	288	428	416	399	217	227	1.07
Second weld	–	–	–	–	–	–	–	–	–	–	–	–	–	0.86
6														
First weld	226	217	411	417	417	254	240	239	392	389	405	218	209	0.98
Second weld	–	–	–	–	–	–	–	–	–	–	–	–	–	1.14

**Table 6:** Comparison of hardness HV10 for different steels for the TBW technique [25,26].

Steel	Ce_IIW_ (%)	HV10max in HAZO	Hardness decrease in HAZO after TBW (pitch)	Fulfillment the criterion of EN-ISO 15614-1:2017 [36] (Lower than 380 HV10)
S355G10 + N	0.385	429	67 (100%)	yes
S460ML	0.365	436	63 (100%)	yes
S460N	0.464	464	54 (87%)	no
